# Role of the Solute-Binding Protein CuaD in the Signaling and Regulating Pathway of Cellobiose and Cellulose Utilization in *Ruminiclostridium cellulolyticum*

**DOI:** 10.3390/microorganisms11071732

**Published:** 2023-07-01

**Authors:** Aurélie Fosses, Nathalie Franche, Goetz Parsiegla, Yann Denis, Maria Maté, Pascale de Philip, Henri-Pierre Fierobe, Stéphanie Perret

**Affiliations:** 1 Campus Scolaire de Coulommiers, Coulommiers, France; 2 Aix Marseille Univ, CNRS, LCB, Marseille, France; 3 Aix Marseille Univ, CNRS, BIP, Marseille, France; 4 Aix Marseille Univ, CNRS, Plateforme Transcriptome, Marseille, France; 5 CNRS, Aix Marseille Univ, UMR7257 AFMB, Marseille, France

**Keywords:** *Clostridium*, solute-binding protein, sensor, regulator, two-component system, cellobiose, cellulose

## Abstract

In *Ruminiclostridium cellulolyticum*, cellobiose is imported by the CuaABC ATP-binding cassette transporter containing the solute-binding protein (SBP) CuaA and is further degraded in the cytosol by the cellobiose phosphorylase CbpA. The genes encoding these proteins have been shown to be essential for cellobiose and cellulose utilization. Here, we show that a second SBP (CuaD), whose gene is adjacent to two genes encoding a putative two-component regulation system (CuaSR), forms a three-component system with CuaS and CuaR. Studies of mutant and recombinant strains of *R. cellulolyticum* have indicated that *cuaD* is important for the growth of strains on cellobiose and cellulose. Furthermore, the results of our RT-qPCR experiments suggest that both the three (CuaDSR)- and the two (CuaSR)-component systems are able to perceive the cellobiose signal. However, the strain producing the three-component system is more efficient in its cellobiose and cellulose utilization. As CuaD binds to CuaS, we propose an in-silico model of the complex made up of two extracellular domains of CuaS and two of CuaD. CuaD allows microorganisms to detect very low concentrations of cellobiose due to its high affinity and specificity for this disaccharide, and together with CuaSR, it triggers the expression of the *cuaABC*-*cbpA* genes involved in cellodextrins uptake.

## 1. Introduction

Cellulose is a renewable material and the most abundant polymer on Earth. Its degradation into mono/oligosaccharides by cellulolytic bacteria is a process of interest for many biotechnological purposes involving the conversion of cellulose into valuable chemicals. Cellulose is made up of chains of glucose units linked through β-1,4 glycosidic linkages. These chains are packed together to form crystalline fibers, making cellulose recalcitrant to enzymatic degradation. In plant cell walls, the release of glucose units from cellulose is therefore a challenging step. Specialized cellulolytic organisms secrete cellulases which allow them to grow on cellulose as their sole source of carbon and energy. In the case of many anaerobic cellulolytic clostridia, cellulases and related enzymes necessary for the degradation of other plant cell wall polysaccharides are gathered in multi-enzymatic complexes called cellulosomes [1]. During the hydrolysis of cellulose, soluble cellodextrins ranging from two (cellobiose) to six (cellohexaose) glucose units are released and have to be taken up by cellulolytic organisms to sustain their growth [2,3,4].

**Figure 1:**
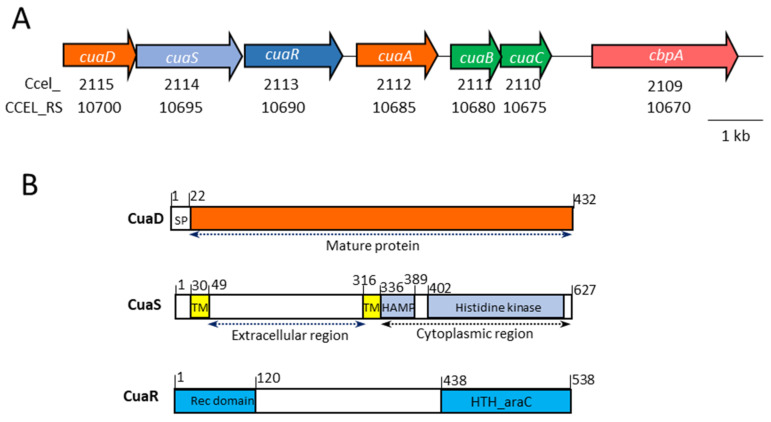
The cluster *cua*. (**A**). Genetic organization. The old and current loci of the genes are indicated below the corresponding gene. (**B**). Organization of the protein domains of CuaD, CuaS, and CuaR. SP, signal peptide; TM, transmembrane helix; HAMP domain present in histidine kinases, adenyl cyclases, methyl accepting proteins, and phosphatases; HTH, helix turn helix domain; Rec domain, response regulator receiver domain. Numbers above correspond to the amino acid number in the sequence of the corresponding protein.

Cellobiose is the major product released by the cellulolytic system of the model cellulolytic bacterium *Ruminiclostridium cellulolyticum* [5]. It is imported, along with other longer cellodextrins, by a unique ATP-binding cassette transporter (ABC transporter) called Cua (cellulose utilization associated) [4]. Cellodextrins are further processed in the cytosol by the cellobiose phosphorylase CbpA and other cellodextrin phosphorylases CdpA, B, and C, generating α-glucose 1-P and glucose, which fuel the glycolytic pathway [4,6]. The CuaABC importer is composed of three proteins: the solute-binding protein CuaA, which binds to cellodextrins with sizes ranging from two to five glucose units, with a *K*_D_ value ranging from 200 to 600 nM respectively, as well as CuaB and CuaC, which assemble to form the transmembrane channel. The ATPase energizing the system is encoded somewhere else in the genome [4,7,8]. CuaABC and CbpA have been found to be essential for the growth of the bacterium on both cellobiose and cellulose-based media [4,6]. The genes *cua* are organized in two operons *cuaABC*-*cbpA* and *cuaDSR* (Figure 1A). The expression of the operon *cuaABC-cbpA* is up-regulated by the regulator CuaR. This is encoded by a gene located in the second operon *cuaDSR*, which is constitutively expressed and located upstream of *cuaABC-cbpA*. *CuaDSR* encodes another solute-binding protein, CuaD, which specifically binds to cellobiose with a higher affinity than that of CuaA (*K*_D_ 20 nM) [4], and two proteins forming a putative two-component system encompassing a sensor (CuaS) and a regulator (CuaR) (Figure 1B).

Two-component systems are sensing and regulating systems triggering cellular responses according to specific environmental stimuli. They are usually composed of a sensor histidine kinase which perceives the signal and activates the cognate response regulator by phosphotransfer. In turn, the regulator induces a cellular response, usually by inducing gene expression which adapts the cell to the environmental conditions. In some cases, an additional protein such as SBP is required for the activation of the sensor of two-component systems or of chemoreceptors [9,10,11,12]. In *R. cellulolyticum*, we previously showed that the inactivation of *cuaD* by the integration of a type-II intron prevented the growth of the mutant on both cellobiose and cellulose [4]. The growth defect is explained by a polar effect due to the introduction of the intron in *cuaD*, lowering the expression of the downstream genes *cuaS* and *cuaR*. The silencing of the signal transduction pathway prevents the production of the ABC transporter and CbpA, making the strain unable to use cellulosic substrates [4]. In the present study, we aim to decipher the sensing system that regulates cellobiose uptake and the degradation in *R. cellulolyticum* by complementing the *cuaD* mutant strain with vectors expressing different genes of the operon *cuaDSR*. It is the first in vivo characterization of a signal transduction pathway dedicated to cellobiose, which is the most common degradation product of cellulose generated by cellulolytic microorganisms.

## 2. Materials and Methods

### 2.1. Strains and Media Vectors

Strains used in this study are reported in Table S1, and vectors and primers are reported in Table S2. *Escherichia coli* strains were grown at 37 °C in Lysogeny broth medium supplemented with appropriate antibiotic (100 µg mL^−1^ of ampicillin or 25 µg mL^−1^ kanamycin). *R. cellulolyticum* H10 ATCC 35319 was grown anaerobically at 32 °C on minimal medium [13], supplemented with appropriate antibiotic (5 µg mL^−1^ thiamphenicol or 2.5 µg mL^−1^ erythromycin) and containing either 2 g.L^−1^ cellobiose or arabinose or 5 g.L^−1^ Sigmacell20 cellulose (Sigma-Aldrich, Saint Louis, MO, USA). After growing the strains on cellobiose- or arabinose-supplemented basal medium, their optical density was monitored at 450 nm over time. When cultured on 5 g.L^−1^ Sigmacell20 cellulose, growth was monitored by measurement of the total protein content as previously described [14].

### 2.2. Transcriptional Analysis

RNA preparation and reverse transcription were performed from *R. cellulolyticum* derivative strains grown in minimal medium supplemented with arabinose (2 g L^−1^) or cellobiose (2 g L^−1^) as previously described [4]. For transcriptional analysis, quantitative real-time PCR was performed using SsoFast EvaGreen Supermix 2X Kit (Bio-Rad, Marnes-la-Coquette, France), and the results were analysed with Bio-Rad CFX Manager software, version 3.0 (Bio-Rad, France) as previously described [4].

### 2.3. Cloning of the Gene Encoding Truncated CuaS_EC_ in E. coli

CuaS_EC_ corresponds to the extracellular domain of CuaS and was designed to be fused in frame with a sequence of 6-histidine residues at its C terminus. Amplification of the gene was performed by PCR using the primer pair ECsenseurNdeDir and ECsenseurXhoRev and genomic DNA of *R. cellulolyticum* as the matrix. The amplicon was then digested with NdeI and XhoI and cloned into an NdeI-XhoI linearized pET22b(+), thereby generating the pET*cuas_EC_*. The plasmid was used to transform BL21(DE3) strain to produce the protein.

### 2.4. Production and Purification of the Recombinant Protein

*E. coli* BL21(DE3) transformed with the pET*cuas_EC_* was grown at 37 °C with shaking. Culture, induction, and purification procedure were performed as previously described [4,15]. The protein was then concentrated and buffer was exchanged with 30 mM Tris HCl with a pH of 8.0 by ultrafiltration (Vivaspin 20, 10 kDa cutoff, Sartorius, Germany). Its final concentration was determined using its specific extinction coefficient.

### 2.5. Complementation of MTLcuaD Strain

For complementation studies, the vectors pSOS*cuaDSR*, pSOS*cuaSR*, and pSOS*cuaR* were constructed from the pSOS956 [4]. Amplicons were obtained by PCR using genomic DNA from *R. cellulolyticum* as the template and the primers pairs 2115BamDir/2113NarRev (*cuaDSR*), 2114BamDir/2113NarRev (*cuaSR*), and 2113BamDir/2113NarRev (*cuaR*). After digestion with BamHI and NarI, the amplicons were ligated to pSOS956 digested with the same enzymes, generating the vectors pSOS*cuaDSR*, pSOS*cuaSR*, and pSOS*cuaR*. These vectors and the pSOSzeroTm [4] were transferred into the MTL*cuaD* mutant strain as previously described [16,17].

### 2.6. Preparation and Western Blot Analysis of Membrane Proteins from R. cellulolyticum

Membrane proteins from wild-type and derivative strains of *R. cellulolyticum* were obtained as previously described [4]. Protein concentration was measured using the Lowry method [18]. After migration using sodium dodecyl sulfate–polyacrylamide gel electrophoresis (SDS-PAGE) and transfer to a nitrocellulose membrane, the membrane proteins were probed with primary antibodies raised against CuaA and CuaD proteins, respectively, as previously described [15].

### 2.7. Isothermal Titration Calorimetry

Ligand binding parameters were measured using a Microcal iTC200 calorimeter (Malvern) at 20 °C in 30 mM Tris HCl at a pH of 8.0, and the result was analyzed as previously described [4].

### 2.8. Bacterial Two-Hybrid Assays

Bacterial two-hybrid assays were performed as previously described [19]. The signal sequence of CuaD and CuaA was deleted, and the extracellular domain of CuaS was fused with either T25 or T18 of the adenylate cyclase from *Bordetella pertussis* at the N or C terminus. The corresponding DNA regions were amplified from genomic DNA using the following primer pairs (Table S2): T25-CuaSEC-PstIdir/T25-CuaSEC-BamHIrev, CuaSEC-T25-PstIdir/CuaSEC-T25-BamHIrev, CuaD-T18-PstIdir/CuaD-T18-BamHIrev, and CuaA-T18-HindIIIdir/CuaA-T18-BamHIrev. Amplicons were then cloned in the vectors pKT25/pKNT25 and pUT18/pUT18C (Hybrigenics, Paris, France) (a generous gift from Dr. L. Journet) using PstI or HindIII and BamHI sites, generating the vectors listed in Table S2. *E. coli* strain BTH101 was transformed with two vectors encoding pairs of proteins to be tested and fused to T18 and T25, respectively. Transformed cells colonies were grown in LB medium supplemented with ampicillin, kanamycin, and isopropyl-*β*-D-thiogalactopyranoside (IPTG) (100 µM) overnight at 30 °C in presence or absence of cellobiose (1%). A total of 5 µL of each culture was dropped onto LB plates containing X-Gal, ampicillin, kanamycin, IPTG (100 µM), and cellobiose when indicated.

### 2.9. Model Building and Docking Experiments

Model building of the monomers and the complexes was performed using the whole sequence of mature proteins of CuaA and CuaD with 429 and 410 residues, respectively, using Alphafold2 and Alphafold2 multimers on the free ColabFold v1.5.2. server, which uses the MMesqs2 server to calculate the multiple sequence alignment. The tetrameric complex of either two CuaA or two CuaD monomers with a dimer of the truncated extracellular domain of CuaS (CuaS_EC_ 267 residues) was calculated using an in-house Alphafold2 server with sufficient GPU memory [20,21]. Ligand docking was performed with the SMINA variant of the Autodock/Vina program under Windows, using an in-house created plugin (GitHub) for the PyMol structure visualization program [22,23]. All ligands were constructed based on monomer fragments from the PDB databank, assembled, and minimized with the free AVOGADRO program [24]. Sequence alignment was performed using Muscle from the EMBL-EBI web service [25].

## 3. Results

### 3.1. Proteins of the Putative Three-Component System

The putative three-component system CuaDSR encompasses a solute-binding protein, a sensor, and a regulator, as depicted in Figure 1. CuaD is predicted to have an N-terminal signal sequence for lipoprotein signal peptidase II, and after maturation, it bears an N-terminal acylated cysteine. This mature form of CuaD is a 410-amino-acid-long solute-binding protein interacting only with cellobiose and not with other longer cellodextrins or glucose. The affinity of CuaD for cellobiose is 10 times higher than that of CuaA for the same disaccharide [4]. CuaS is a class-I sensor histidine kinase of 627 amino acids. It is predicted to be an integral membrane protein spanning the membrane through two transmembrane helices (the stretch of amino acids from 30 to 49 and 316 to 339) delimitating an extracytosolic section with no predicted domain. CuaS also contains a HAMP domain (HAMP domain is present in histidine kinases, adenyl cyclases, methyl accepting proteins, and phosphatases) and a histidine kinase domain, both of which are predicted to be in the cytosol (Figure 1). The soluble form of the extracytosolic part of the sensor (CuaS_EC_) was successfully produced in *Escherichia coli* and purified. Its ability to bind to cellulose degradation products was analyzed by isothermal titration calorimetry. This revealed that CuaS_EC_ has no affinity for arabinose, cellobiose, or cellotetraose.

CuaR is a response regulator of 538 amino acids belonging to the YesN/AraC family, which contains a response regulator receiver domain with a conserved phosphorylation site (D55) at the N terminus and an HTH_AraC regulatory helix-turn-helix domain at the C terminus. In the protein, a stretch of 318 amino acids without any predicted domain or function can be found between the receiver domain and the HTH_AraC domain. CuaR has been previously shown to induce the expression of the operon *cuaABC-cbpA* [4].

### 3.2. Overproduction of CuaR in cuaD Mutant Strain

We previously constructed a mutant of *R. cellulolyticum* (MTL*cuaD*) in which the gene *cuaD* was interrupted by a type-II intron. This mutant was unable to grow on cellobiose- or cellulose-based media. To study in vivo the three-component transduction pathway, we verified first whether the overproduction of the regulator CuaR alone would complement the phenotype of the mutant strain. We therefore transformed the *cuaD* mutant strain with the vector expressing the *cuaR* alone. Normally the regulator cannot be activated without its cognate sensor. However, when present in large quantities, a regulator might trigger its own activation even in the absence of its cognate sensor. This might be due to possible unspecific dimerization events or because of possible cross-talk with other sensor(s) [26].

**Figure 2:**
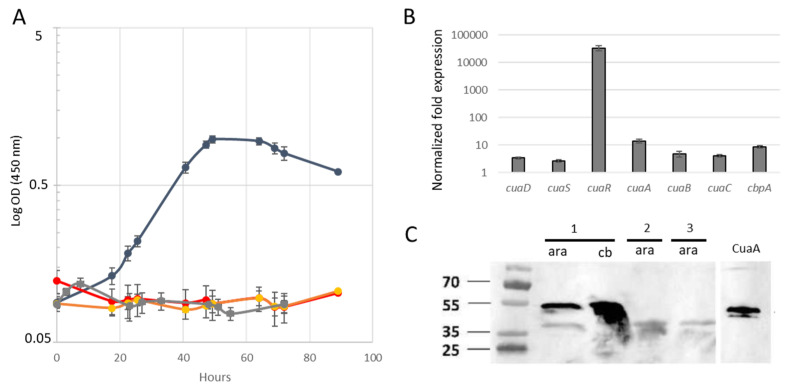
Analysis of the mutant strain transformed with pSOS*cuaR*. (**A**). Growth of the strains on cellobiose-containing minimal medium: wild-type (dark blue), MTL*cuaD* (red), MTL*cuaD* (pSOS-empty) (orange), and MTL*cuaD* pSOS*cuaR* (grey). (**B**). Normalized fold expression of the genes of the *cua* cluster in the strain MTL*cuaD* pSOS*cuaR* compared to mutant strain in an arabinose-containing minimal medium. (**C**). Western blot analyses of membrane fractions extracted from the wild-type strain (1), MTL*cuaD* strain (2), and MTL*cuaD* pSOS*cuaR* strain (3) using antibodies targeting CuaA. Membrane fractions with protein amounts of 20 µg or 4 µg extracted from strains cultivated in a medium containing arabinose or cellobiose, respectively, were loaded on the SDS-PAGE.

In the presence of arabinose as the sole carbon source (used as a control sugar), the mutant strain MTL*cuaD*(pSOS*cuaR*) grew as fast as the wild-type or the control mutant strain MTL*cuaD*(pSOSzeroTm), indicating that the overexpression of *cuaR* did not inhibit its growth (Figure S1). However, when using a cellobiose-based medium, the mutant strain that expressed the regulator in trans did not grow, as was the case for the mutant MTL*cuaD* and the control mutant strain MTL*cuaD* (pSOSzeroTm), thus showing that the overexpression of *cuaR* cannot restore the growth of the mutant strain on cellobiose (Figure 2A). The expression of the operon *cuaABC-cbpA* remains at a basal level in this strain, though the gene *cuaR* is efficiently expressed in the complemented strain (Figure 2B). The lack of CuaA was confirmed in this strain by Western blot analyses (Figure 2C). These results suggest that the regulator overproduced from the pSOS*cuaR* failed to induce the expression of the operon *cuaABC-cbpA* in the absence of the cognate sensor and is therefore not sufficient to restore the strain’s growth on cellobiose.

### 3.3. Role of CuaD in the Signaling System

As the overproduction of the regulator did not allow the mutant strain to grow on cellobiose, further complementation experiments with genes encoding the regulator with the sensor and/or the SBP CuaD were performed to examine the role of CuaD in the three-component system. The *cuaD* mutant strain hosting vectors harboring either the genes *cuaSR* or *cuaDSR* was grown in minimal medium containing arabinose. Both strains displayed similar growth and doubling times compared with those of the wild-type, MTL*cuaD*, or MTL*cuaD*(pSOSzeroTm) strains (Figure S1), thus indicating that the presence of the vectors did not negatively impact the fitness of the strains.

**Figure 3:**
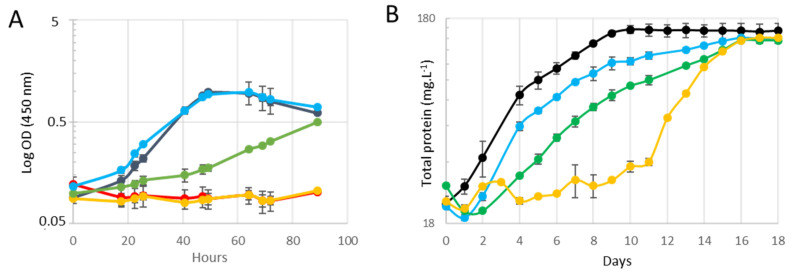
**Growth of different strains of *R. cellulolyticum***. Strains were grown in minimal medium containing 2 g L^−1^ cellobiose (**A**) or 5 g L^−1^ cellulose (**B**). The strains are wild-type (black), MTL*cuaD* (red), MTL*cuaD*(pSOSzeroTm) (yellow), MTL*cuaD*(pSOS*cuaSR*) (green), and MTL*cuaD*(pSOS*cuaDSR*) (blue). Experiments were performed in triplicate, and bars indicate the standard deviation.

The *cuaD* mutant strain containing either the vector pSOS*cuaDSR* or pSOS*cuaSR* was grown on cellobiose or cellulose as the growth substrate (Figure 3). Both strains expressing multi-component systems were able to grow on cellobiose, in contrast to the mutant strain which was only producing the regulator alone. Complementation with the complete operon *cuaDSR* fully restored the growth phenotype as compared to the wild-type strain, whereas complementation with the two-component system allowed the mutant strain to grow at a much slower rate, reaching an OD of 0.5 only after 90 h of culture (Figure 3A). The mutant strain, as well as the mutant strain transformed with the empty pSOS vector, did not grow at all, as previously observed [4]. On the cellulose, the *cuaD* mutant strain carrying the empty pSOS vector started to grow after 10 days because of an adaptation of the strain which at least restored the production of CuaA as previously described [4]. The mutant strain transformed with the pSOS*cuaDSR* or pSOS*cuaSR* displayed similar growth on the cellulose; however, the mutant strain producing the three-component system grew faster than the strain producing only the two-component system (Figure 3B).

**Figure 4:**
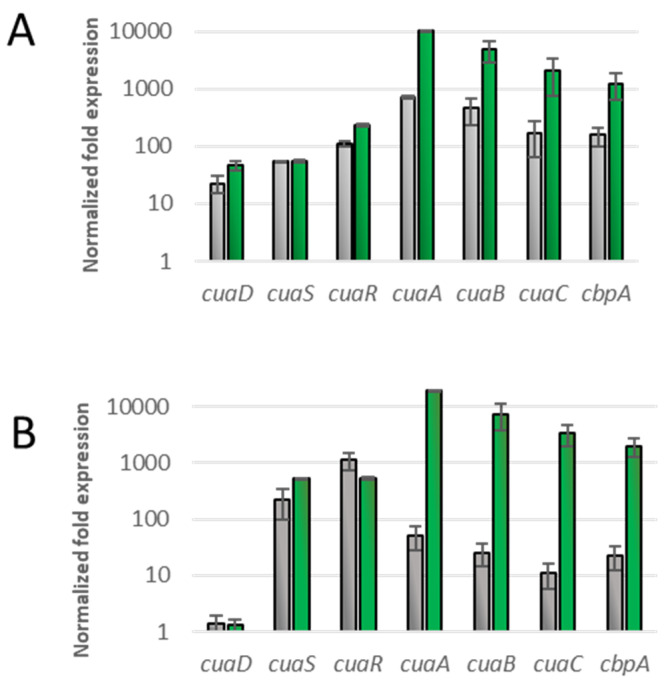
qPCR analysis of mRNA of *cua* genes. The analyses were made on derivatives of *R. cellulolyticum* mutant strains MTL*cuaD*: MTL*cuaD*(pSOS*cuaDSR*) (**A**) or MTL*cuaD*(pSOS*cuaSR*) (**B**). Total RNA was extracted from cultures of the strains grown to OD 0.6 in minimal medium supplemented with 0.2% arabinose (gray bars) or 0.2% cellobiose (green bars) as the only carbon source. Normalization was performed using the expression level of RNA16S gene. Normalized fold expression was calculated comparatively to the strain MTL*cuaD* grown in minimal medium containing 0.2% arabinose. Error bars indicate the standard deviation of three independent experiments.

**Figure 5:**
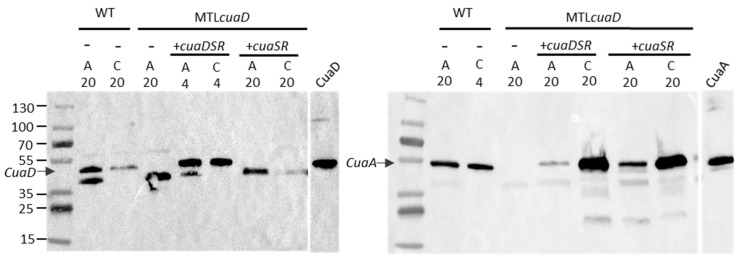
Detection of CuaD and CuaA in *R. cellulolyticum* derived strains. Membrane fractions were isolated from wild-type strain (WT) cells, as well as mutant-strain MTL*cuaD*, MTL*cuaD*(pSOS*cuaDSR*), and MTL*cuaD*(pSOS*cuaSR*) cells grown in minimal medium supplemented with 0.2% arabinose (A) or 0.2% cellobiose (C). Either 4 or 20 µg was loaded on the SDS-PAGE, as indicated on the top of the gel. The (-) sign means that no vectors are present. The detection was performed with antibodies probing CuaD (**left**) or CuaA (**right**). Pure recombinant proteins CuaA (0.3 µg) and CuaD (0.01 µg) were loaded as positive controls. Arrows indicate the location of CuaD and CuaA.

To further analyze their phenotype, we studied the expression level of *cuaDSR* and *cuaABC-cbpA* in these complemented strains cultivated either with arabinose or cellobiose (Figure 4). The expression level of the genes of both operons was analyzed when the cultures reached an OD of 0.6 with cellobiose as the growth substrate and was normalized with the expression level of the genes in the strain MTL*cuaD* cultivated in a medium containing arabinose. As expected, *cuaD* was expressed only in the strain complemented with the pSOS*cuaDSR*, whereas *cuaS* and *cuaR* were expressed in both types of complemented strains. In both strains, the expression of the genes *cuaABC* and *cbpA* was induced 10 to 100 times more with cellobiose as the substrate compared to the arabinose culture, showing that cellobiose induces *cuaABC-cdpA* expression in both strains. When grown on arabinose, the increase in the expression level of *cuaABC-cbpA* was higher in the strain MTL*cuaD*(pSOS*cuaDSR*) than that in MTL*cuaD*(pSOS*cuaSR*). One possible explanation is that the lack of CuaD in the signaling system allows a better locking of the signaling pathway. This could also be due to the overproduction of the signaling system from the pSOS vector (high-copy vector) that disturbs the sensitivity of the pathway. The results also suggest that both the three- and the two-component systems can sense the signal (cellobiose) and induce the expression of *cuaABC*-*cbpA*. The Western blot analysis confirmed that CuaD is only present in the strain overproducing CuaD and that CuaA is produced in the strains complemented with either pSOS*cuaDSR* or pSOS*cuaSR*, but it seems to be more abundant in the presence of cellobiose than in the presence of arabinose (Figure 5).

### 3.4. Binding of SBPs with CuaS

**Figure 6:**
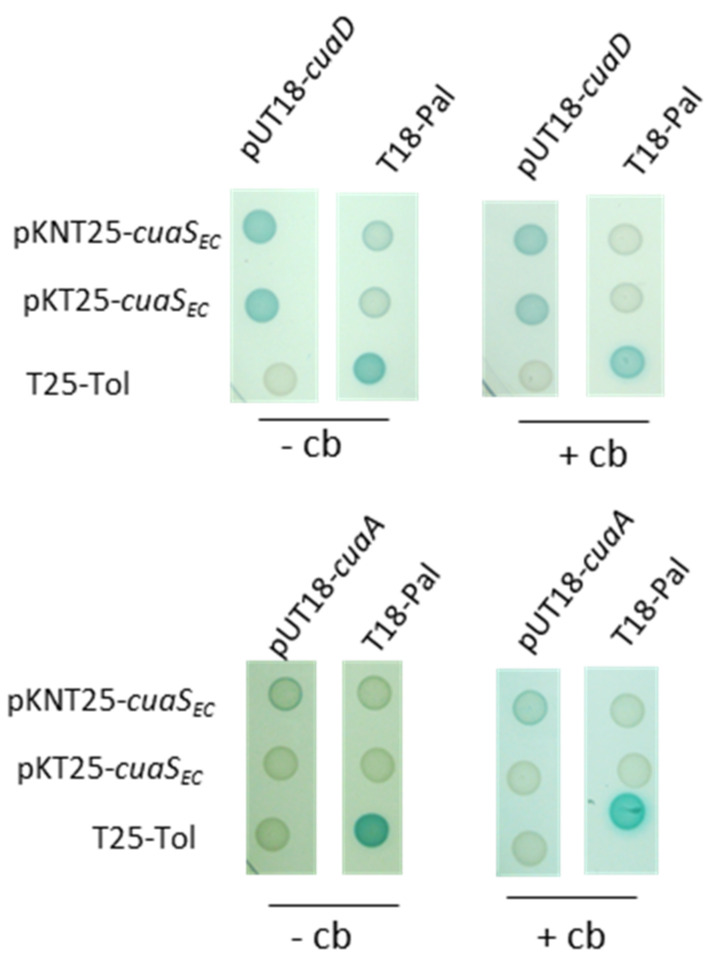
Binding test of CuaS with CuaD or CuaA by bacterial two-hybrid system. The BTH101 strain containing a combination of vectors derived from pUT18/pUT18C and pKT25/pKNT25 was spotted on agar plate supplemented with IPTG, X-Gal with or without 0.1% cellobiose (Cb). The Tol/Pal interaction constitutes the positive control. *cuaS_EC_* expressed the extracellular part of CuaS.

To gain further insights into the signaling pathways, we tested the ability of the sensor to bind either CuaD or CuaA by using a bacterial two-hybrid system (BATCH) (Figure 6). The DNA region encoding the two solute-binding proteins without their signal peptide and the extra-cytoplasmic domain of the sensor CuaS_EC_ were fused to the N or the C terminus of the complementary fragments T18 and T25 of the adenylate cyclase. Together, they reconstitute the catalytic domain of the adenylate cyclase. Independently of the presence or absence of cellobiose in the culture medium, CuaS_EC_ was found to bind to CuaD but not to CuaA (Figure 6).

### 3.5. Model of the Quaternary Structure

We constructed models of the monomers CuaA and CuaD, as well as the tetrameric complexes CuaD/CuaS_EC_ and CuaA/CuaS_EC_ using Alphafold2. Based on the previously solved structure of the quaternary complex of XylFII-LytSN containing two SBPs and two extracellular domains of the sensor LytS [27], our complex models were made up of two SBPs with two CuaS_EC,_ called AASS (CuaA–CuaA–CuaS_EC_–CuaS_EC_) and DDSS (CuaD–CuaD–CuaS_EC_–CuaS_EC_). The best calculated monomeric models of CuaA and CuaD had a high average individual confidence index (pLDDT) above 90, except for the N-terminal region, which may have been disordered (res 1–20). Their two-domain structure resembles other Family 1 SBP structures [28,29].

**Figure 7:**
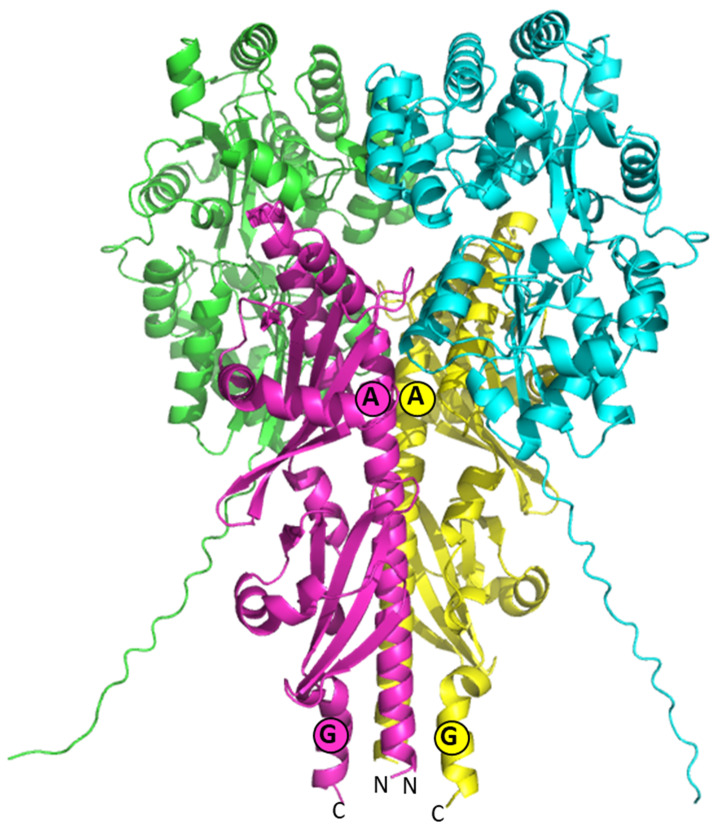
Alphafold model of the complex CuaD–CuaD–CuaS_EC_–CuaS_EC_ (DDSS). The SBP CuaD are in green and cyan while CuaS_EC_ are in yellow and pink. CuaS_EC_ contains 7 helices, with letters from A to G. Circled letters of the same color correspond to CuaS_EC_ monomer.

The predicted model of the CuaS_EC_ dimer is a rod-like structure whose backbone is formed by two long N-terminal helices (letter A), each coming from one monomer. These central helices (A) are each decorated by two five-stranded antiparallel beta sheets, which each form a kind of α–β–α sandwich with an additional alpha helix (D and E). Two C-terminal helices (F) form a four-helix bundle with the central helices at the N-terminal side which constitutes the bottom of the rod, while a pair of two helices (B and C) that are connected by an Asn-rich loop form a cap on the upper beta sheet of each side on the top of the rod. Helix C is one of the main interactors with the CuaD monomer in the complex, while the Asn-rich loop contains a sequence of five asparagine residues in a row which may be disordered and is the most uncertain part of the model. In both models of the complex structures, the pLDDT values of each monomer are quite good, ranging from 82.8 to 85.9% (CuaS_EC_) and from 84.7 to 87.9% (CuaA or CuaD). However, only the DDSS model has a reasonable pTM value of 0.79, while the interface in a modeled AASS complex is poorly packed with a pTM value of 0.64. This supports our biochemical experiments which failed to detect any interaction between CuaA and CuaS_EC_. The best DDSS model is presented in Figure 7. In the CuaD/CuaS_EC_ model, both domains of the SBPs are in close contact with one sensor domain.

**Figure 8:**
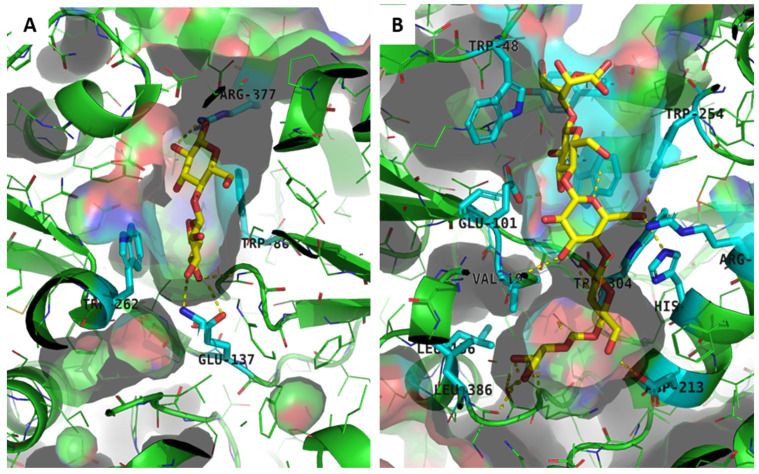
Molecular docking of CuaD and CuaA models. (**A**) Best-scoring result of cellobiose in CuaD model obtained by molecular docking. The cellobiose is colored yellow. The molecular docking of the complex CuaD–cellobiose reveals hydrophobic stacking with Trp262 and hydrophobic head interaction with Trp86 (in cyan). H-bonding interaction is observed with Lys36, Glu137, and Arg377 (also in cyan). (**B**) Best-scoring result of cellopentaose in CuaA model obtained by molecular docking. The cellopentaose is colored yellow. A longer pocket is observed with multiple hydrophobic interactions involving Trp48, Trp254, Tyr279, Trp275, Trp384, and Phe104 in the upper part and Trp304, Val102, Leu386, and Leu136 at the bottom of the pocket. H-bonding interactions are observed with the main chain around Ser385, Asp213, Glu101, Arg206, and His210. Visible residues in cyan are underlined.

We previously reported different substrate specificities between CuaD and CuaA towards cellodextrins [4]. CuaD binds only to cellobiose with a high affinity (*K*_D_ 20 nM), whereas CuaA binds to a larger range of cellodextrins that might contain two to five glucose units, with *K*_D_ values ranging from 200 to 600 nM, respectively. To better understand these differences in specificities, we performed a virtual docking of the cellobiose and cellopentaose with the CuaA and CuaD monomers. It was shown that long cellodextrin molecules, such as cellopentaose, can fit in a large groove in the CuaA model but not in the more restricted binding pocket in the CuaD model (Figure 8). In the latter, only cellobiose could be docked (Figure 8). The best-scoring cellobiose positions occupied the same binding pocket as observed in a solved X-ray structure of the complex of a xylotriose with a similar SBP XBP1 sequence from *Caldanaerobius polysaccharolyticus* (pdb code: 4G68, sequence identity: 28.3%) (Figure S3) [30]. As observed for the published xylotriose/SBP complex, the cellobiose molecule establishes hydrophobic stacking interactions with aromatic amino acids, but due to an amino acid insertion in a helix in CuaD and the structural rearrangement of the following loop at the entrance of the binding pocket, this pocket is too small to fit dextrins longer than disaccharides. In contrast to that in CuaD, aromatic stacking is not observed in the center pocket of CuaA but in the groove part leading to the center pocket. These models match the experimental data previously obtained by ITC [4].

## 4. Discussion

In summary, our results indicate that the three-component system is better adapted for the utilization of cellobiose and cellulose than the incomplete two-component system. Indeed, the absence of CuaD in the signaling system of the mutant strain containing the vector pSOS*cuaSR* leads to the slower growth of the strain on both cellobiose and cellulose. The continuation of the growth of this strain might be due to the expression of *cuaABC* and *cbpA*, which remains possible even in absence of CuaD, as revealed by the qRT-PCR and Western blot results. The expression of these genes could be explained by the overproduction of CuaS and CuaR, which might disturb the signaling pathway. Another explanation is the sensing of cellobiose by the sensor directly, as shown by the analysis of the expression level of the *cua* genes in the transformed mutant strains. However, this interaction between CuaS and the cellobiose is probably weak, and the signal transmission seems to be less efficient than that with the participation of CuaD. This was shown by the growth results of the recombinant strains producing the complete system.

CuaD, CuaS, and CuaR thus form a three-component system in which the interaction of CuaD with its ligand triggers a regulation event through its interaction with CuaS. Several three-component systems, including a solute-binding protein (SBP) that interacts with a two-component system, have been described so far [9,10,11,12,31,32]. Usually the binding of the ligand to the SBP activates the sensor, causing the upregulation of the targeted genes [10,11,12,27,31,32]. In some of these systems, the SBP forms a heterocomplex with the sensor. The binding of the ligand to the SBP either induces a conformational change in the heterocomplex [12,33], dissociates the complex [11], or assembles two SBP/sensor heterodimers into a heterotetramer complex (two SBP/two sensors) [27]. We showed that in the CuaDSR system, CuaS interacts with CuaD in the presence or absence of cellobiose and might form a tetrameric complex. This is also supported by our modelling studies. We hypothesize that the binding of cellobiose to CuaD triggers a conformational change in the sensor, inducing the phosphorylation cascade and *cuaABC-cbpA* gene induction.

Among the three-component systems reported in the literature, four of them control the expression of genes encoding components of transporters [10,11,31,32,33]. In the system dedicated to chitin utilization in *Vibrio cholera* and citrate import in *Bordetella pertussis*, only one SBP is encoded which is involved in both transport and signaling [11,33]. In two other systems reported in *Clostridium beijerinckii* and *Geobacillus stearothermophilus*, both of which manage carbohydrate import, two SBPs are encoded as is the case for the *cua* system of *R. cellulolyticum* [10,31]. The two SBPs have distinct functions: one is for sensing, and the other is for substrate uptake. In the Cua system, CuaD has a high affinity (*K*_D_ of 20 nM) and high specificity to cellobiose. CuaA (the SBP for transport), on the other hand, binds to cellobiose and longer cellodextrins with sizes up to that of cellopentaose with lower affinity. These differences are supported by our modelling studies. Further information on ligand/protein interactions may be obtained in future molecular dynamics studies. With CuaD assisting CuaS, this three-component system might specifically detect very low concentrations of cellobiose and induce the expression of the genes dedicated to the import and metabolization of cellodextrins through the action of the regulator CuaR. Cellodextrins are efficiently imported by the CuaABC transporter during their growth with cellulose [4]. This efficient import leaves only a low concentration of cellobiose, which might still be detectable by CuaD/S, helping to maintain the induction of *cuaABC-cbpA* genes during growth. Cellobiose induces the expression of the operon *cuaABC-cbpA*, which is consistent with the fact that cellobiose is the most abundant degradation product of cellulose released by cellulosomes [5]. In addition, it is the smallest degradation product of cellulose to indicate to the bacterium its presence in the environment. The same is not true for glucose, which can also be released from other polysaccharides, such as amylose or glucomannan.

## Data Availability

The datasets used and/or analyzed during the current study are available from the corresponding author on reasonable request.

## Supplementary Materials

The following supporting information can be downloaded at: https://www.mdpi.com/article/10.3390/microorganisms11071732/s1, Figure S1: Growth of strains derived from *R. cellulolyticum*; Figure S2: Protein staining of membrane fractions from different strains of *R. cellulolyticum*; Figure S3: Multiple sequence alignment of the sequence of the SBP XBP1 from *Caldanaerobius polysaccharolyticus* with CuaD from *Ruminiclostridium cellulolyticum*. Table S1: Bacterial strains and vectors used, Table S2: Primers used.
