# Supplementary material for: Role of the Solute-Binding Protein CuaD in the Signaling and Regulating Pathway of Cellobiose and Cellulose Utilization in Ruminiclostridium cellulolyticum
Source: Microorganisms. 2023 Jul 1;11(7):1732. doi: 10.3390/microorganisms11071732 (PMC10384115; doi:10.3390/microorganisms11071732)
Supplement: Supplementary file 1 [file microorganisms-11-01732-s001.zip › microorganisms-2458470-supplementary.pdf]

Table S1. Bacterial strains and vectors used

Strain or plasmid	Relevant characteristics	Source or reference
E. coli DH5a	F^- *endA1 hsdR17*(rK^- mK^+) *supE44 thi-1* λ^- *gyrA96 relA1Δ*(*lacZYA argF*) *U169* (Φ80 *lacZΔ*M15) *recA*	Roche Diagnostics
E. coli BTH101	*F-, cya-99, araD139, galE15, galK16, rpsL1 (Str r), hsdR2, mcrA1, mcrB1*	[19]
E. coli SG13009(pREP4)	F^- *his pyrD Δlon-100 rpsL* (pREP4)	Qiagen
E. coli BL21(DE3)	F^- *ompT hsdS* (rB^- mB^-) *gal dcm* (DE3)	Novagen
R. cellulolyticum H10	Wild-type, ATCC35319 DSM 5812	DSMZ
R. cellulolyticum MTL*cuaD*	ATCC35319, *cuaD* ::intron, Erm^r	This study
pET22b(+) and pET28a(+)	*E. coli* expression vector, Amp^r and Kan^r respectively	Novagen
pET*cuaD*	pET22b+ derivative carrying the NdeI-XhoI fragment encoding mature CuaD	[4]
pET*cuaA*	pET28a+ derivative carrying the NdeI-XhoI fragment encoding mature CuaA	[4]
pET*cuaS*$_{EC}$	pET22b+ derivative carrying the NdeI-XhoI fragment encoding extracellular domain of CuaS	This study
pMTL007*cuaD*	pMTL007 derivative targeting *cuaD* at the locus CCEL_RS10700 (old locus Ccel_2115)	Ref Fosses
pSOSzeroTm	*E. coli/Clostridium* shuttle vector (ColE1, pIM13); Ap^r, Cm^r/Tm^r	[15]
pSOS954	*E. coli/Clostridium* shuttle vector (ColE1, pIM13); Ap^r,Em^R, containing an expression cassette controled by the *thiolase* gene promotor (P_{thl}) from *C. acetobutylicum*	[15]
pSOS956	pSOSzero-Tm derivative carrying the SalI-SalI expression cassette from *C. acetobutylicum* from pSOS954, Ap^r, Cm^r/Tm^r	[4]
pSOS*cuaDSR*	pSOS956 carrying the BamHI-NarI fragment encoding full length *cuaA*, *cuaB*, *cuaC* genes, Ap^r, Cm^r/Tm^r	This study
pSOS*cuaSR*	pSOS956 carrying the BamHI-NarI fragment encoding full length *cuaA*, *cuaB*, *cuaC*, *cbpA* genes, Ap^r, Cm^r/Tm^r	This study
pSOS*cuaR*	pSOS956 carrying the BamHI-NarI fragment encoding full length *cbpA*, Ap^r, Cm^r/Tm^r	This study
pUT18	*ColE1* replicon, encoding T18 domain of adenylate cyclase from *Bordetella pertussis*, AmpR	Hybrigenics
pUT18-CuaD	pUT18 derivative encoding CuaD at the N-term of T18 domain	This study
pUT18-CuaA	pUT18 derivative encoding CuaA at the N-term of T18 domain	This study
pKT25	P15A replicon, encoding T25 domain of adenylate cyclase from *Bordetella pertussis*, KanR	Hybrigenics
pKT25-CuaS$_{EC}$	pKT25 derivative encoding CuaS$_{EC}$ at the C-term of T25 domain	This study
pKTN25	P15A replicon, encoding T25 domain of adenylate cyclase from *Bordetella pertussis*, KanR	Hybrigenics
pKTN25-CuaS$_{EC}$	pKT25 derivative encoding CuaS$_{EC}$ at the N-term of T25 domain	This study

Ap^r, ampicilline resistance ; Erm^r, erythromycin resistance ; Kan^r, kanamycine resistance ; Cm^r/Tm^r, chloramphenicol/thiamphenicol resistance

Table S2. Primers used

EXPERIMENT	NAME	SEQUENCE	FEATURES
**Production in *E. coli***	CuaS$_{EC}$NdeID	AATTCAT**ATG**CACCACCACCACCACCACAAGAGTTCTGAAGAAATTATTAACAG	NdeI is underlined, ATG boldface
	CuaS$_{EC}$XhoIR	AATTCTCGAGTTATATCTTGTTTCTCAATTTATTTACATCC	XhoI is underlined
**Complementation**	2115BamDir	TTAAGGATCCAAATAAGAATTGGAGTGAAACAAATGCAAAG	BamHI is underlined
	2113NarRev	AATTGGCGCCTTAATAACAACTATCTCTGTATTCCGAGG	NarI is underlined
	2114BamDir	TTAAGGATCCGTTTATTAGGATATGGTGGAAGAATGATAAAC	NarI is underlined
	2113 BamDir	ATTAAGGATCCAGAATTTAAAAGGAGGGATTAAAATGTACAAGGTTCTAATAATTGATGATG	NarI is underlined
**BATCH**	T25-CuaSEC PstI dir	TGGCGCGCACGCGGCGGGCTGCAGGGAAGAGTTCTGAAGAAATTATTAACAG	PstI is underlined
	T25-CuaSEC BamHI rev	TTAGTTACTTAGGTACCCGGGGATCCTTATATCTTGTTTCTCAATTTATTTACATC	BamHI is underlined
	CuaSEC-T25 PstI dir	GATTACGCCAAGCTTGCATGCCTGCAGGAAGAGTTCTGAAGAAATTATTAACAG	PstI is underlined
	CuaSEC-T25 BamHI rev	AATTCGAGCTCGGTACCCGGGGATCCTCTATCTTGTTTCTCAATTTATTTACATCC	BamHI is underlined
	CuaD-T18 PstI dir	TACGCCAAGCTTGCATGCCTGCAGGAGCAGTGACAATTCGTATG	PstI is underlined
	CuaD-T18 BamHI rev	GAGCTCGGTACCCGGGGATCCTCCTGCAATTTGTCTAATGCTTTTTCC	BamHI is underlined
	CuaA-T18 HindIII dir	AGCTATGACCATGATTACGCCAAGCTTGGGAAACACAGAGTCAGG	HindIIIis underlined
	CuaA-T18 BamHI rev	GAGCTCGGTACCCGGGGATCCTCCTTCAATTCAGGAACATTTTTCTT	BamHI is underlined

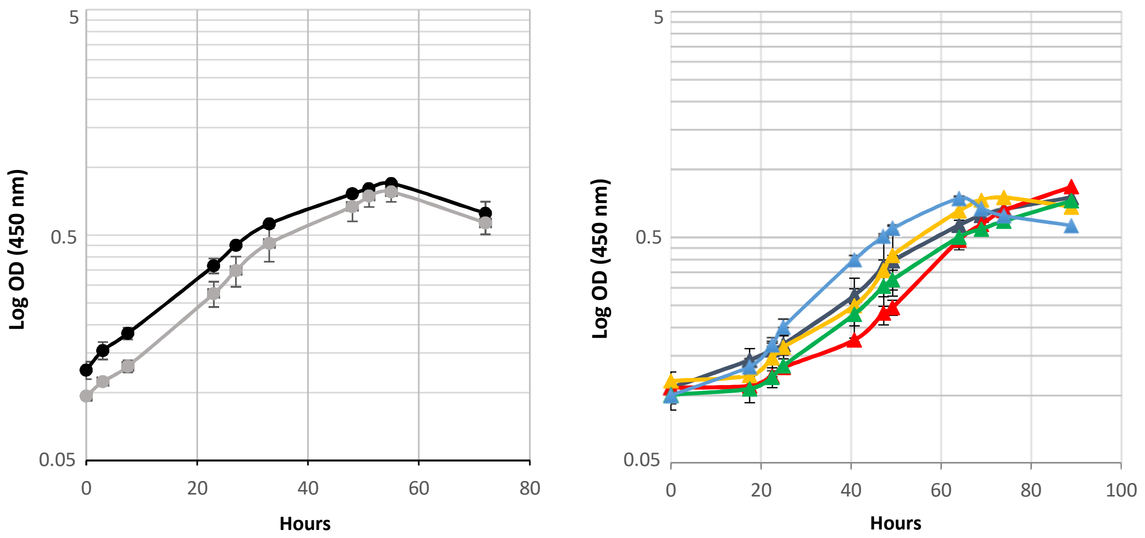

Figure S1. Growth curve of different strains of *R. cellulolyticum*. Strains are grown in minimal medium containing 2 g L^{-1} arabinose. The strains are WT (black), MTL*cuaD* (red), MTL*cuaD*(pSOSzeroTm) (yellow), MTL*cuaD*(pSOS*cuaSR*) (green), MTL*cuaD*(pSOS*cuaDSR*) (blue), MTL*cuaD*(pSOS*cuaR*) (grey). Experiment were performed in triplicate and bars indicate the standard deviation.

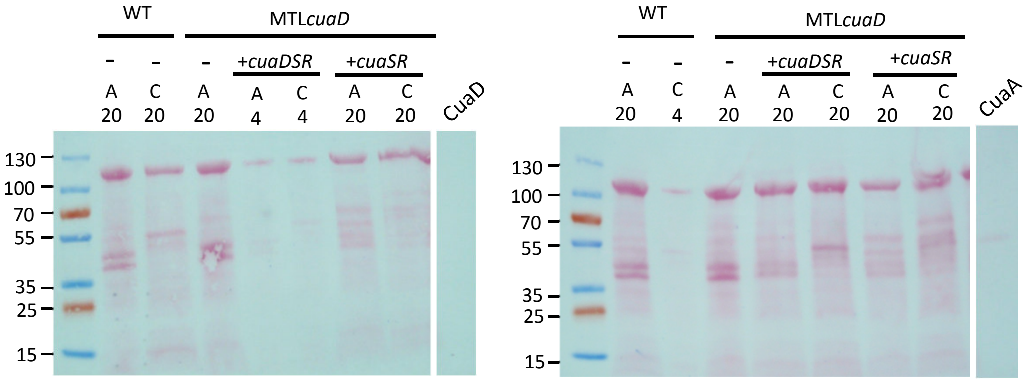

Figure S2. Protein staining of membrane fraction from different strains of *R. cellulolyticum with* Red Ponceau. Membrane fractions were isolated from cells grown in minimal medium supplemented with 0.2% arabinose (A) or 0.2% cellobiose (C) and either 4 or 20 µg was loaded on the SDS-PAGE, transferred onto a nitrocellulose membrane and then stained with Ponceau S red solution. Pure recombinant protein was loaded as positive control CuaA (0.3 µg) and cuaD (0.01 µg). The strains are the wild-type strain (WT), the mutant strain MTL*cuaD*, the strain MTL*cuaD*(pSOS*cuaDSR*) and MTL*cuaD*(pSOS*cuaSR*).

```
CLUSTAL multiple sequence alignment by MUSCLE (3.8)

XBP1      MAHHHHHHVDDDDKMCSSNNLSKSNTSNSSKTSSSSKKMCSSNNLSKSNTSNSSKTSSSS
CuaD      ---------------------------------------CSSDNSYVSNARNSKTTQAET
                                                 ***:*   **: **..*.:.:

XBP1      KKITL-TFWNLFTGEPAKTK-VKEIIDQWNKENPNVQIVESVTENDAYKTKIKAAIAANE
CuaD      STISLMTSWG---GVDSKAGCLMDLLDRFENGNPSIKVSNQSIFGDEYLPTLKTRFASGN
          ..*:* * *.   *  :*:  : :::*.::: **.::: :.   .* * ..:*: :*:.:

XBP1      APDIFQTWAGGFSQPFVEAGKVLQLDSYLNDGTK--DQLLPGSFDNVTYNGKIYGIPFDQ
CuaD      EPDVFGLWPCSDIKYMIMANKLADLTDMLTKDSEWMDSFKGNYFDLTTYNNRIYGIPFEL
           **:*  *. .  : :: *.*: :* . *...::  *.:  . ** .***..******:

XBP1      QASVLYINKELFDKYNVKVPTTFSELIDAIKTFKSKGVTPFALGEKDEWPGMWYY-DMIA
CuaD      VFEGMFINKDLFQQFNVKIPQNYEELKNAINIFNKHNITPIAYNATAE--GSYIYQNMIA
            . ::***:**:::***:* .:.** :**: *:.:.:**:* . .  *  * : * :***

XBP1      LREGGVQLTRDALNGKASFDNQAFTDAAQKLQDMVNAGAFDSGFMGLTRDEATAEFNQGK
CuaD      SLGGNDGVENYMVNNQI---NKCYIDAMKYMKELHKMHAFPTDLISITSEERNNLFIKKQ
             *.  : .  :*.:    *:.: ** : :::: :  ** :.::.:* :* .  * : :

XBP1      AAMYFGGNFDAAAFVSDPSSLVKGKIEAVRFPTIEGGKGDPTEYIGGTVGALMVSANS--
CuaD      AAMIVQGSWFAAYF-----GKFDKTVEMIPFPSMGNGNRKIPAGLGG--GTFYISKSAWG
          ***  . *.: ** *      . .. .:* : **:: .*: . .  :**  *:: :* .:

XBP1      --KYKDEAVRAAKYLAKQLSDMDYLI-ATGLPAWKYDNIDQSKVDPLE------IQIMNN
CuaD      TPNSKENTVKLLKFLTSKETS-DYLYKESGL----FSTLNISRETPFNALAKQSIDIYEN
            : *:::*.  *:*:.: :. ***   :**    :..:: *.  *::      *:* :*

XBP1      IVANAKGSVPAWDIYLSGDAAQTHKDLVAQLFAKQITPEEYSKQMQQKINGK--
CuaD      TPEQDRCAIPDHVI-----DRSTWEKIIVKEFPDYLDDKISAEQIWEKALDKLQ
             : . ::* . *        .*.:.::.: *.. :  :  ::*:.:*  .*
```

Figure S3. Multiple sequence alignement of the sequence of the SBP XBP1 from *Caldanaerobius polysaccharolyticus* with CuaD from *Ruminiclostridium cellulolyticum*. The alignment was made by Muscle multialignment tool available at EMBL-EBI web services (25)
